# Detection of newly produced T and B lymphocytes by digital PCR in blood stored dry on nylon flocked swabs

**DOI:** 10.1186/s12967-017-1169-9

**Published:** 2017-04-05

**Authors:** Marion Vaglio Tessitore, Alessandra Sottini, Aldo M. Roccaro, Claudia Ghidini, Simona Bernardi, Giovanni Martellosio, Federico Serana, Luisa Imberti

**Affiliations:** Centro di Ricerca Emato-oncologica AIL (CREA), ASST Spedali Civili, P.le Spedali Civili, 1, 25123 Brescia, Italy

**Keywords:** Digital PCR, Flocked swabs, KRECs, TRECs

## Abstract

**Background:**

A normal number of T-cell receptor excision circles (TRECs) and K-deleting recombination excision circles (KRECs) is considered a biomarker for adequate new T- and B-cell production. In newborns, detection of TRECs and KRECs by real time PCR from dried blood spotted on filter paper is used for the screening of severe immunodeficiency. In adults, elderly and during diseases, where the number of TRECs is lower than in newborns and children, a large amount of DNA and a sensitive method of amplification are necessary to identify newly produced lymphocytes.

**Methods:**

DNA was prepared from blood of 203 healthy adults (range: 18–91 years old) absorbed for 10 s on flocked swabs and let to dry, or from peripheral blood mononuclear cells. DNA was subjected to digital PCR and to well established conventional real time PCR-based method using TREC- and KREC-specific primers and probes. The number of TRECs and KRECs was expressed per mL of blood. Statistical analysis was performed by nested ANOVA, Pearson coefficient of determination, and by linear regression tests.

**Results:**

The novel method for the storage of dried blood on nylon flocked swabs and the use of digital PCR allow quantification of TRECs and KRECs with high degree of sensitivity, specificity, accuracy, and precision. TRECs and KRECs were amplified by digital PCR in all tested blood samples, including those obtained from elderly individuals (>70 years old) and that were negative by real time PCR. Furthermore, values of TRECs and KRECs obtained by digital PCR were in the range of those acquired by real time PCR.

**Conclusions:**

Our findings demonstrate that DNA isolation from dried blood on flocked swabs followed by digital PCR-based analysis represents a useful tool for studying new lymphocyte production in adults and elderly individuals. This suggests the potential use of the methodology when monitoring of clinical variables is limited by the number of molecules that can be amplified and detected, such as in patients with immunodeficiency or under immunosuppressive therapies.

**Electronic supplementary material:**

The online version of this article (doi:10.1186/s12967-017-1169-9) contains supplementary material, which is available to authorized users.

## Background

T-cell receptor (TCR) and B-cell receptor gene rearrangements involve cutting and splicing of the DNA encoding the alternate variable, diversity, and joining segments. Excised DNA, formed during the programmed gene rearrangements in the thymus and in the bone marrow, constitutes the TCR excision circles (TRECs) and K-deleting recombination excision circles (KRECs), respectively [1, 2]. These circles are stably retained within peripheral blood lymphocytes, are not replicated during mitosis and are diluted as T and B cells proliferate. The number of TRECs and KRECs per unit of blood volume correlates with the production of new T and B cells; a normal TREC and KREC number is considered a biomarker for a good thymic and bone marrow output, while low or absent TRECs or KRECs indicate poor T- or B-cell production [1, 2]. TRECs have been measured starting from different biological materials, such as fresh whole blood (WB) [3], peripheral blood mononuclear cells (PBMC) [1, 4] or dried blood samples [5]. TRECs and KRECs were initially measured in separate quantitative real time PCR (qRT-PCR) assays [1, 2]; successively, we modified these assays to allow the simultaneous measure of TRECs and KRECs in PBMC (“reference” method) [6, 7], while others used the method to measure the target molecules on dried blood spotted on filter paper cards [8, 9]. TREC/KREC combined assay has been used to establish appropriate diagnosis and treatments of patients with severe primary immune deficiencies [7, 10, 11], to monitor immune-reconstitution in patients receiving chemotherapy and post-hematopoietic stem cell transplantation [12], or undergoing immunomodulant [13] and anti-HIV [14] therapies. The protocol performed using dried blood spots is currently used for newborn screening of immunodeficiency [8, 9, 15], considering the high number of TRECs in newborns, the low cost of the test, and the easy transportation and storage of spotted cards. The use of dried blood samples on filter paper prepared from adults, elderly people, and patients undergoing immunosuppressive therapies may be challenging due to the low quantity of TRECs and KRECs that can be isolated and the low quantity of DNA that can be extracted by punches of cards. The recovered DNA quantity represents a critical factor for the success of TREC and KREC testing. Generally, the more DNA is recovered, the better is the chance for obtaining a robust and reliable result; on the contrary, a small amount of DNA would result either in stochastically variable amplification or in the loss of signal due to insufficient template.

Digital PCR (dPCR) is a novel, alternative method to qRT-PCR for precise quantification of nucleic acids, enabling many applications that require high sensitivity and have restricted sample availability. In dPCR, the specimen is distributed across a large number of partitions, each of them containing one (or a few) or no copies of the target DNA. By counting the number of partitions containing (positive partitions) or not (negative partitions) the amplified target PCR product, the absolute abundance of the target molecule can be measured in a given sample without the need of the standard curve required in the qRT-PCR assay. There are mainly two types of platforms for dPCR: one incorporating a microfluidic chip and one in which the PCR is performed in microdroplets [16]. They have many potential applications, including the detection of low-level molecules [17], rare genetic sequences [18], circulating miRNAs [19], copy number variations [20], as well as gene levels in single cell [21].

In this work, we describe the feasibility of processing blood samples absorbed on flocked swabs (FS), dried, and stored at room temperature for subsequent DNA isolation, followed by TREC and KREC quantification using the Quantstudio^®^ 3D Digital PCR, a micro-well chip-based dPCR platform, for the measure of new T-and B-cell production.

## Methods

### Sample collection and storage

Anonymized blood samples were obtained from patients with immunodeficiency and from adult controls (CTRL) without pathologies referring to the ASST Spedali Civili of Brescia hospital for routine diagnostic testing. Blood samples were collected using ethylenediaminetetraacetic acid (EDTA) tubes. Two types of nylon FS (FLOQSwab™ Genetics, Copan, Brescia, Italy) were tested: a single FS with an integrated active drying system in the tube stopper (4N6FLOQSwab code 4504C) and a dual FS without the drying system (4N6FLOQSwab code 4511C). All FS were immersed in blood for a time ranging from 10 to 60 s; the first type of FS was immediately inserted into containers, while the other FS, without the integrated drying system, were left to dry for at least 2 h and then inserted back into containers. FS were stored at room temperature until DNA extraction. For sample stability evaluation, DNA was extracted after 1 week, and then after 1, 3, 6, 9 and 12 months from FS preparation.

Blood aliquots were also used to prepare PBMC by Ficoll-Hypaque gradient centrifugation which were kept in liquid nitrogen; other aliquots were directly subjected to DNA extraction. Simultaneously, fresh blood drops were placed on Nucleic-Cards™ (Copan Italia, Brescia, Italy), which were stored at room temperature until processing. Discs of 2 mm were punched from each card and subjected to DNA extraction. DNA extracted from HeLa cell line, which does not contain TRECs or KRECs, was used to assess dPCR specificity and to establish background signals.

### DNA extraction

DNA from FS was extracted either with QIAamp DNA Blood Mini Kit and with Gentra Puregene Blood Kit according to manufacturer’s instructions (Qiagen, Hilden, Germany). Red blood cell lysis was not performed with the Gentra kit. In addition, we developed the homemade protocol for DNA extraction as detailed below. FS shafts were snapped at the molded breakpoint, inserted in the Nucleic Acid Optimizer (NAO™) basket (Copan), placed in microtubes and filled with 300 µL of saline solution, 300 µL of ATL buffer and 7 µL of Puregene Proteinase K 20 mg/mL (Qiagen). After an overnight incubation at 56 °C on a shaker at 1400 rpm, samples were centrifuged at 13,000 rpm for 5 min. During this centrifugation the grid bottom of the basket allows the passage of sample elution buffer from the FS. FS and baskets were removed from the microtubes and protein precipitation was obtained by incubating samples on ice for 20 min with 233 µL of ammonium acetate 7.5 M. After centrifugations at 13,000 rpm for 10 min, supernatants were transferred to new microtubes containing 600 µL of isopropanol and 1 µL of 5 mg/mL glycogen (Ambion Inc., Austin, TX), mixed gently until DNA was visible as threads or clumps, and then incubated for 10 min at room temperature. Vortexing was avoided to minimize fragmentation of the extracted DNA [22]. After centrifugation at 13,000 rpm at 4 °C for 20 min, supernatants were discarded and pellets washed with 500 µL of ethanol 70%, incubated at room temperature for 5 min and centrifuged at 13,000 rpm at 4 °C for 10 min. Dried pellets were subsequently dissolved using 40 µL of TE (10 mM Tris, pH 8.0, 0.1 mM EDTA, pH 8) and incubated at 65 °C for 10 min on a shaker.

DNA from 300 µL of WB and from six 2-mm punch discs of dried blood on cards (corresponding to 8.4 µL of blood) was extracted using the Gentra Puregene Blood Kit following the manufacturer’s instructions. DNA from 3 × 10^6^ of PBMC and HeLa cell line was extracted using the QIAamp DNA Blood Mini Kit.

DNA from FS and cards without absorbed blood were used to test eventual cross contaminations during extraction procedures.

### Quantity, purity, and integrity of DNA extracted from FS

DNA extracted from FS, cards, WB, PBMC, and Hela cells was analyzed spectrophotometrically (Nanodrop 2000c, Thermo Fisher Scientific, Waltham, MA) at 260 nm to evaluate DNA concentration and by measuring the 260/280 nm absorbance ratio to estimate DNA purity.

The level of precision of the homemade method for DNA extraction from FS was determined in several replicates by evaluating intra- and inter-assay variation. Yield was expressed as percentage of recovery that is the proportion of extracted over expected DNA. The expected DNA was calculated based on the white blood cell (WBC) count and a mean DNA content of 6.6 pg/cell.

For DNA integrity and size range analysis, 500 ng of DNA were subjected to electrophoresis on a 0.8% agarose gel in 1× TBE buffer (0.089 M Tris base; 0.089 M boric acid; 0.002 M EDTA, disodium salt dihydrate; final pH 8.3) containing ethidium bromide. The DNA size marker was Lambda DNA *Hin* *d* *III* Digest (Sigma-Aldrich, Saint Louis, MO). To further evaluate genomic DNA integrity and to exclude the presence of any inhibitory material interfering with amplification reaction, 50 ng of DNA were amplified in a 50 μL mix containing 500 nM of primers specific for a fragment of albumin gene (forward: 5′-TGA AAC ATA CGT TCC CAA AGA GTT T-3′ and reverse: 5′-CTC TCC TTC TCA GAA AGT GTG CAT AT-3′), 0.2 mM deoxynucleotide triphosphates, 1.5 mM MgCl_2_, and 1.5 U AmpliTaq Gold DNA polymerase with reaction buffer II (Applied Biosystem™, Austin, TX). A no-template control was used to check for contamination. The amplification program consisted of one step at 95 °C for 7 min, followed by 30 cycles of 95 °C for 30 s, 60 °C for 30 s, 72 °C for 30 s and a final extension at 72 °C for 10 min. Amplified products were separated on 2% agarose gel containing ethidium bromide; the DNA size marker was DNA molecular weight marker VIII (0.019–1.11 kbp; Roche Diagnostics, Basel, Switzerland). Gels were photographed by G:Box™ gel documentation system (Syngene, Cambridge, UK).

### Quantification of TRECs and KRECs

TRECs and KRECs were measured after amplification with both TaqMan qRT-PCR and dPCR assays. The well established “reference” qRT-PCR was carried out as previously reported, using DNA extracted from PBMC, with a triple plasmid containing fragments of TRECs, KRECs, and TCR constant alpha gene created in our laboratory and used to prepare the standard curve [7]. With this method, the number of lymphocytes plus monocytes (which are the cells obtained in PBMC preparation) contained in 1 mL of blood was used to calculate the number of TRECs or KRECs per milliliter of blood (copies/mL), that is = (TRECs or KRECs per 1 × 10^6^ PBMC) × (lymphocyte plus monocyte count in 1 mL of blood)/10^6^ [7].

dPCR was performed using micro-well- chip-based QuantStudio^®^ 3D Digital PCR (Applied Biosystems™, Thermo Fisher Scientific, Waltham, MA), following the manufacturer’s instructions and the same primers and probes for the TaqMan assay, with the exception of 5′ reporter dye of KREC probe labelled with VIC instead of HEX. Four microliter of DNA (corresponding to about 500 ng depending on sample concentration) were added to a mixture consisting of 8 µL of 2× QuantStudio^®^ 3D Digital PCR master mix v2, 900 nM of both TREC and KREC forward and reverse primers and 200 nM of both TREC FAM-TAMRA and KREC VIC-TAMRA probes, and 0.29 μL of BSA (2 mg/mL). Using automatic chip loader, 14.5 μL of this mix were added on each QuantStudio^®^ 3D Digital PCR 20K Chip v2. Chips were loaded on ProFlex™ 2× flat PCR System and cycled according to the following parameters: 95 °C for 8 min, followed by 45 cycles of 62 °C for 1 min and 95 °C for 15 s, and a final extension at 62 °C for 2 min.

Singleplex dPCR was performed amplifying 4 μL (500 ng) of DNA in a mixture consisting of 8 µL of 2× QuantStudio^®^ 3D Digital PCR master mix v2, 900 nM of forward and reverse primers for TRECs (or KRECs) and 200 nM of probe for TRECs FAM-TAMRA (or KRECs VIC-TAMRA), and 0.29 μL of BSA (2 mg/mL).

Absolute quantification was determined using Quantstudio^®^ 3D Digital PCR System and analyzed with QuantStudio^®^ 3D AnalysisSuite Cloud Software (Thermo Fisher Scientific).

Sensitivity and specificity of dPCR platform were evaluated with the plasmid created for qRT-PCR diluted in the DNA from HeLa cell line. The copy number was estimated according to the molecular weight, amount, and length of plasmid. Plasmid dilutions were set up in 125 ng/µL of DNA from HeLa cells; the plasmid copy numbers charged on chip were 0, 5, 10, 25, 50, 100, 500, 1000, and 2500 and the amount of charged DNA was 500 ng. The sensitivity of dPCR was compared with that of qRT-PCR by testing, with the two methods, progressive dilutions of plasmid DNA copies.

The reproducibility of dPCR assay was determined by intra- and inter-assay variation experiments, performed starting from the DNA obtained from FS prepared using the same blood sample.

### Direct quantification of TRECs and KRECs per mL of blood

When amplified DNA is extracted from blood, either in liquid form or absorbed and dried onto a FS, TRECs and KRECs per mL of blood can be calculated according to the following equation, which takes into account the variable recovery of DNA due to an inevitable DNA loss during extraction:

$$\left[ {{\text{Target}}/(\upmu{\text{L}})} \right] = {\text{QTY}}_{\text{target}} \times \frac{{{\text{DNA}}_{\text{extracted}} ({\text{ng}})}}{{{\text{DNA}}_{\text{loaded }} \left( {\text{ng}} \right)}} \times \frac{1}{{{\text{Vol }}\left( \upmu{{\text{L}}} \right)}} \times \frac{1}{\text{Rec}}$$

where: QTY_target_ = copy number of target sequence in the loaded DNA, as determined by qRT-PCR or dPCR (in the case of dPCR, this is the copy number per chip, obtained by multiplying the copy number per partition by the number of filled partitions), DNA_extracted_ (ng) = total amount of extracted DNA determined after measuring the DNA concentration using the spectrophotometer, DNA_loaded_ (ng) = actual amount of DNA loaded for the PCR reaction, Vol (μL) = volume of WB used for DNA extraction from blood in liquid form or absorbed onto a FS, Rec = proportion of DNA recovered after the extraction.

Rec can be calculated as:

$${\text{Rec}} = \frac{{{\text{DNA}}_{\text{extracted}} \left( {\text{ng}} \right)}}{{{\text{DNA}}_{\text{tot}} \left( {\text{ng}} \right)}}$$

The DNA_tot_ (ng) is the theoretical total DNA content in the liquid blood volume used for the extraction/blood absorbed onto the FS, and can be estimated as follows:

$${\text{DNA}}_{\text{tot}} \left( {\text{ng}} \right) = {\text{WBC}}/\left( \upmu {{\text{L}}} \right) \times {\text{Vol}}\left( \upmu{{\text{L}}} \right) \times {\text{DNA}}_{{1{\text{cell}}}} ({\text{ng}})$$

where: DNA_lcell_ = mean DNA content in human cells (6.6 × 10^−3^ ng).

After substituting these terms in the first equation, we obtain the following formula:

$$\left[ {{\text{Target}}/(\upmu {\text{L}})} \right] = {\text{QTY}}_{\text{target}} \times \frac{{{\text{WBC}}/\upmu ({\text{L}}) \times 6.6 \times 10^{ - 3} ({\text{ng}})}}{{{\text{DNA}}_{\text{loaded}} \left( {\text{ng}} \right)}}$$

Alternatively, because 1 mL = 10^3^ μL, we can directly obtain [Target/(mL)] as:

$$\left[ {{\text{Target}}/({\text{mL}})} \right] = {\text{QTY}}_{\text{target}} \times \frac{{{\text{WBC}}/(\upmu {\text{L}}) \times 6.6}}{{{\text{DNA}}_{\text{loaded}} \left( {\text{ng}} \right)}}$$

where the final estimation of the number of targets depends neither on the exact volume collected by the FS (which varies in distinct FS), nor on the estimation of DNA recovery; it only depends on the amount of loaded DNA and on the number of WBC/mL of blood, assuming that the absorption process does not alter the concentration of cells present in the sample (i.e. absorbed blood WBC concentration being equal to the circulating blood WBC concentration).

### Statistics

The percentage of DNA recovery was analyzed by nested ANOVA, thus accounting for the presence of replicates and for the different days in which the experiments were performed. The variance components estimated by the nested ANOVA were employed to calculate the coefficients of variation, following the Clinical and Laboratory Standards Institute EP05-A3 document suggestions (http://shop.clsi.org/method-evaluation-documents/EP05.html). Correlations were analyzed with the Pearson coefficient of determination. TREC and KREC changes with age were assessed by linear regression. Results were considered significant if p < 0.05.

## Results

### Blood absorption on FS

FS immersed into blood achieved the maximum quantity of absorption, and subsequent plateau, within 10 s. At this time point, the mean volume of absorbed blood, measured by quantifying the weight of blood contained in the tube prior to introduction and after removal of the FS, was 143 μL (range: 117–160 μL). No differences were found if FS with or without the integrated active drying system in the tube stopper were used (data not shown). However, for safety reasons, all the following experiments were performed on FS with the drying system. Indeed, before being inserted into containers, FS without active drying system needed to be air-dried for at least 2 h on the desk. Furthermore, the immediate insertion of swabs with active drying system into containers prevents potential microbial growth.

### Quantity and quality of recovered DNA

We first examined the extraction method yielding the greatest quantity of DNA from dried blood absorbed on FS. The mean amount of DNA per μL of elution volume and total DNA yield, recovered for each extraction method performed on dried blood of the same CTRL absorbed on FS, is shown in Table 1.

**Table 1:** Extraction efficiency of DNA from the flocked swabs impregnated with whole blood using three different extraction methods

Extraction method	Elution volume (µL)	Mean DNA concentration (ng/µL)	Standard deviation (ng/µL)	Mean of total DNA yield (ng)	Standard deviation (ng)
QIAamp DNA Blood Mini Kit	25	18.00	1.74	450	87.18
Gentra Puregene Blood Kit	30	92.75	6.73	2783	201.80
Homemade protocol	40	121.07	8.53	4843	341.39

Experiments were done in triplicate

Data indicated that the optimized homemade method, as described within the material and method section, allowed the maximum yield of DNA recovered from FS, and was therefore used for validation experiments of dPCR method. No differences were found if DNA was extracted from CTRL (126.57 ± 10.49 ng/µL) or from patients (123.68 ± 22.24 ng/µL). The mean concentration of DNA recovered with this protocol from CTRL-derived FS was 26.3 ng/μL of absorbed blood (range: 19.3–36.9 ng/μL). Of note, the concentration did not change if DNA extraction was done 1 week, or 1, 3, 6, 9 and 12 months after FS preparation (data not shown). The mean concentration, obtained from mean of absorbed blood of 143 μL, was comparable to that obtained starting from 300 μL of WB (mean: 25.2 ng/μL of blood; range: 21.2–29.1 ng/μL) and from six 2-mm punch discs obtained from filter paper cards (mean: 26.8 ng/μL of blood; range: 23.2–57.1 ng/μL). The mean percentage of DNA recovery (i.e. proportion of extracted over expected DNA) from the three sources was comparable (p = ns), being 69% (95% CI 60–78%) for FS, 60% (49–70%) for WB, and 74% (62–85%) for cards.

**Fig. 1:**
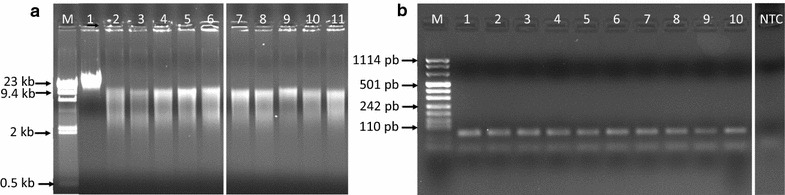
DNA integrity and size range assessed by agarose gel electrophoresis. **a** *Lane M* DNA high molecular weight marker, *lane 1* positive control (DNA extracted from whole blood), *lanes 2*–*6* 5 DNA prepared from the same blood sample and absorbed on different FS, and *lanes 7*–*11* DNA of 5 different blood samples absorbed on FS. **b** *Lane M* DNA molecular weight marker, *lanes 1–10* 10 μL of albumin-amplified products of DNA extracted from different FS samples, *lane NTC “no template control”* DNA sample extracted from FS without absorbed blood

DNA integrity, size range, and detectability by PCR were measured by gel electrophoresis. FS-derived DNA showed a smear-like profile, with peak intensity always greater than 1000 base pairs (Fig. 1a), which is accepted as a minimum size range for several genetic and epigenetic studies [22]. After PCR amplification, extracted DNA exhibited on agarose gel a clearly recognizable 82 base pair band of the albumin housekeeping gene (Fig. 1b). This demonstrated the quality of the isolated DNA and the absence of inhibitory substances. Furthermore, the amplification of this short fragment indicated that the DNA prepared from FS was suitable for the amplification of TRECs and KRECs, leading to amplified products of 88 and 90 base pairs, respectively. Intra- and inter-assay coefficient of variation for DNA recovery from FS, were 13.1% (95% CI 9.9–19.4%) and 13.6% (5.7–36.7%), respectively, as calculated in 26 replicates.

### Validation of dPCR for TREC and KREC quantification

DNA was subjected to dPCR as described within the Material and Method section. The substitution of the HEX fluorochrome with VIC at 5′ end of KREC probe in respect to that used for the TaqMan assay improved both the fluorescence intensity and the separation between cluster of negative and positive data points. Quality control of chip-based dPCR was performed with a software which assesses whether the data on a chip are reliable based upon loading, signal, and noise characteristics. In order to obtain a precise quantification, the threshold was settled at 15,000 data points.

TREC- and KREC-free DNA extracted from HeLa cells was used to assess the specificity of dPCR. HeLa DNA resulted positive for 1 TREC/chip and 3 KRECs/chip, which can be considered as a background signal (Additional file 1: Figure S1A). HeLa-generated data were also used to establish the threshold values, manually set at fluorescence intensity of 5000 for TRECs and 4000 for KRECs. These threshold values allow for a precise separation between positive and negative dot plots (Additional file 1: Figure S1B).

**Fig. 2:**
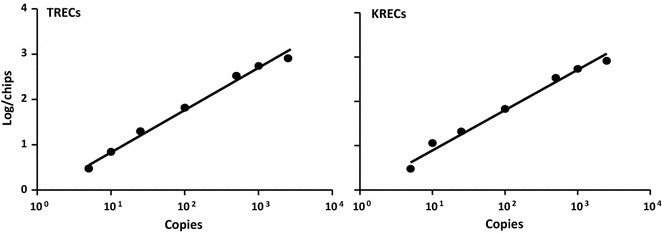
Sensitivity of dPCR for TREC and KREC amplification. Zero, 5, 10, 25, 50, 100, 500, 1000, and 2500 copy number of the plasmid containing fragments of TRECs and KRECs were diluted in HeLa cell DNA to obtain 500 ng of DNA and then subjected to dPCR for TREC and KREC quantification

Sensitivity of dPCR in detecting TRECs and KRECs was evaluated amplifying known quantities of the plasmid DNA containing the sequences of TRECs and KRECs, diluted in the HeLa cell DNA. When increasing plasmid copies were loaded per chip, we found a high correlation between plasmid copy numbers determined by dPCR and the estimated ones (r^2^ = 0.9903 for TRECs and 0.9848 for KRECs; Fig. 2).

Experiments performed using several dilutions of TRECs- and KRECs-containing plasmid demonstrated that the detection limit was one copy of TRECs and one of KRECs with dPCR, while 2.5 copies of the target molecules were detected by qRT-PCR. However, with qRT-PCR approach, the lowest levels of TRECs and KRECs are evidenced at cycle thresholds (Ct) greater than 35, which approaches the sensitivity limits of the qRT-PCR system. Both digital and qRT-PCR gave rise to undetectable results at 0 copies of TREC and KREC plasmid.

The efficiency of multiplex dPCR was assessed by comparing the number of TRECs and KRECs obtained with duplex (TRECs: 5614/mL ± 117, and KRECs: 30,468/mL ± 3230) and singleplex (TRECs: 4509/mL ± 208 and KRECs: 26,595/mL ± 2912) reactions.

Intra-assay variation of dPCR was 11% for TRECs and 8% for KRECs, while inter-assay variation was 13% for TRECs and 15% for KRECs (Additional file 2: Table S1). These data are in agreement with published results obtained using the established “reference” qRT-PCR based assay [7, 12].

### dPCR for TREC and KREC quantification

As first step, we quantified TRECs and KRECs using the “reference” qRT-PCR method assessed on PBMC-derived DNA [7], and subsequently we selected 10 samples with increasing number of TRECs (from undetectable to 30,817/mL) and KRECs (from undetectable to 22,099/mL). The samples with undetectable/low levels of both targets were obtained from children with primary immunodeficiency, while those with higher levels of TRECs and KRECs were from CTRL. For these samples, WB- and FS-absorbed-DNA samples also were available. Of note, dPCR allowed the detection of TRECs and KRECs in all analyzed samples, even in those in which they were undetectable with qRT-PCR (subject #1, #2, #3 and #4; Table 2).

**Table 2:** Values of TRECs and KRECs

Subject	Source of DNA	TRECs/mL qRT-PCR	TRECs/mL dPCR	KRECs/mL qRT-PCR	KRECs/mL dPCR
	PBMC^a^	und	–	8294	–
#1	WB	und	261	17,636	16,684
	FS	und	74	17,183	15,041
	PBMC	3674	–	und	–
#2	WB	7854	6564	und	1312
	FS	7892	8488	und	1488
	PBMC	18	–	1136	–
#3	WB	252	462	4880	4048
	FS	und	106	2739	3580
	PBMC	149	–	6897	–
#4	WB	und	527	10,670	13,244
	FS	und	405	11,330	14,249
	PBMC	5220	–	7279	–
#5	WB	11,999	8231	22,300	17,112
	FS	4467	8046	17,494	15,230
	PBMC	5322	–	25,159	–
#6	WB	10,221	4824	41,282	32,753
	FS	4817	6403	31,934	28,184
	PBMC	9544	–	17,189	–
#7	WB	39,062	33,378	66,717	16,582
	FS	18,071	15,147	61,276	40,361
#8	PBMC	19,264	–	4357	–
	WB	27,926	23,385	7088	3096
	FS	27,828	22,671	3619	3959
#9	PBMC	24,809	–	23,333	–
	WB	25,778	25,811	20,599	17,346
	FS	24,563	20,244	26,541	16,852
#10	PBMC	30,817	–	22,099	–
	WB	52,494	38,190	42,171	32,453
	FS	41,428	35,845	42,037	35,649

*dPCR* digital PCR, *FS* flocked swabs, *PBMC* peripheral blood mononuclear cells, *qRT-PCR* quantitative real time PCR, *und* undetectable, *WB* whole blood

^a^DNA from PBMC was analyzed with the “reference” method

Furthermore, although performed on three differently manipulated blood specimens (PBMC, WB and dried blood on FS), with DNA prepared with three different DNA extraction methods (QIAamp and Gentra Puregene Blood Kit, and homemade protocol), using two different types of quantitative PCR, and done in several different experimental procedures, results obtained from each different subject were similar, in particular in the samples in which TRECs and KRECs were lower than 10,000/mL. TRECs and KRECs were also evaluated by normalizing data of different experimental procedures of dPCR for the amount of DNA prepared from FS and directly from WB. With few exceptions, the values of TRECs and KRECs per μg of DNA obtained with the two techniques were comparable (data not shown).

Then, 5 replicate aliquots of samples, chosen from those with undetectable number of TRECs or KRECs at the qRT-PCR assay and from which sufficient DNA was available, were analyzed in different experimental procedures both by dPCR and qRT-PCR. Results demonstrated that TRECs and KRECs remained constantly undetectable by qRT-PCR, but were always detectable by dPCR (Additional file 3: Table S2).

**Fig. 3:**
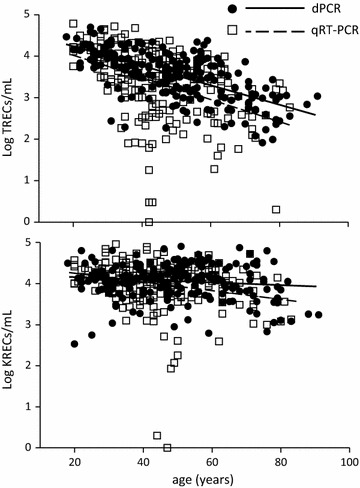
Levels of TRECs and KRECs in adults. *Black round dots* and *continuous lines* results obtained by digital PCR (dPCR) starting from DNA obtained from dried blood adsorbed on FS; *square dots* and *dotted lines* results obtained by quantitative real time PCR (qRT-PCR) starting from DNA prepared from peripheral blood mononuclear cells

Subsequently, dPCR was used to quantify TRECs and KRECs on 203 FS-absorbed blood samples from CTRL of different age (from 18 to 91 years old); the results were compared with values of TRECs and KRECs obtained with the “reference” method in 207 age-matched CTRL (from 20 to 83 years old). The age-adjusted mean value (±standard error) of TRECs was 7383 ± 553/mL (range: 82–49,929/mL) when tested with dPCR, and 6535 ± 548/mL (range: 1–61,171/mL) when tested with qRT-PCR (p = ns). The age-adjusted mean value of KRECs was 16,834 ± 1023/mL (range: 341–81,634/mL) with dPCR vs 15,117 ± 1015/mL (range: 0–92,180) with qRT-PCR (p = ns). Figure 3 shows the perfect overlap of the results achieved with the two methods. Furthermore, the data obtained by dPCR confirmed that the levels of KRECs were stable over time (slope = −23 [95% CI −144 to 99], p = ns), while TRECs progressively decreased with increasing age (slope = 264 [95% CI −319 to −208], p < 0.0001). Although present at very low levels in the group of 30 subjects >70 years old (Additional file 4: Figure S2), TRECs were detected in all samples analyzed with dPCR.

## Discussion

In this study we demonstrated that quantity and quality of DNA prepared from dried blood stored on FS are adequate to quantify circularized fragments of DNA, such as TRECs and KRECs, even if they are contained in peripheral blood in number as low as few copies per mL. Therefore, the proposed strategy for storing dried blood on FS and TREC and KREC quantification by dPCR can be successfully applied to adults and elderly subjects for a proper evaluation of new T- and B-lymphocyte generation.

It is well known that blood samples adsorbed on filter paper or nylon FS, and left to dry, represent a convenient alternative option to fresh blood specimens when collection and shipment of biological materials are difficult, when resources are limited, or if used for applications in large surveys. Indeed, dried blood carries a reduced biohazard risk as compared to liquid samples; it simplifies shipments to reference laboratories for testing, and importantly, it requires minimal storage facilities because nucleic acids are stable at room temperature. For these reasons, dried blood spotted as a drop onto filter paper is largely used in newborn screening for genetic diseases [23]. The most recent improvement within this specific field of research is the newborn screening for severe combined immunodeficiency (SCID) through the detection of TRECs. In fact, the use of TREC test was introduced for early diagnosis of infants with SCID, following the evaluation of incidence and costs of early versus late treatment [24, 25]. Several protocols for easy detection of TRECs in newborn blood spotted on filter paper have been developed and validated [24, 26–28]. The absolute number of TRECs varies greatly, with several fold-differences, ranging from 119 to 1900 TRECs/μL, based on different laboratories [25], depending on the parameters of the assays, such as DNA isolation method, qRT-PCR primers and probes, standard curve used, singleplex or multiplex PCR. The volume of total dried blood could be an important limiting factor leading to assay failure when, for instance, the biological substrates are contained at very low quantity and if a large representation of target molecules is mandatory. Indeed, while normal newborns have a high rate of new T-cell production, resulting in TREC numbers of about 10% of their total T-cell numbers, adults have progressively lower ratio of TRECs to T cells, reflecting peripheral T-cell expansion [1]. Therefore, due to the lower representation of TRECs within their peripheral blood, several punches from filter paper would be required in order to obtain a sufficient DNA amount for TREC quantification. To our knowledge, data regarding the values of TRECs in the blood of adults spotted on filter paper are not available. In addition, those obtained from liquid and dried blood samples or PBMC [1, 3, 4, 29] are of difficult comparison due the methods of TREC calculation or because TREC measurement has been presented in a number of different ways. Indeed, TRECs are reported as normalized amount (calculated as a difference between Ct values of reference gene and Ct of TRECs), as absolute number per unit number of cells, as TREC molecules per μg of DNA within PBMC or T-lymphocyte subsets, or as TRECs per mL of blood. The values that we have generated by directly quantifying TRECs from FS-derived dried blood amplified by dPCR alternative method are given per mL, are measured removing any confounding attributable to cell proliferation, and using very small quantities of starting whole blood (about 150 μL). The possibility to store dried blood on FS and to send it by regular mail (Guidance on Regulations for the Transport of Infectious Substances 2015–2016—WHO/HSE/GCR/2015.2) lends this approach to be easily used in clinical settings where limited tissue is available, for example in adult and ageing studies, in immunodeficient patients and/or where longitudinal information is required, and when the analysis cannot be performed locally.

We found that the extent of age-related TREC decrease determined by dPCR in DNA obtained from dried blood adsorbed on FS was comparable to that reported by Lorenzi et al. [3], stating a 1.5-Log change of TRECs over the age range they have measured in liquid WB. Furthermore, newly produced data can be compared with retrospective ones because the mean values of dPCR-derived TRECs and KRECs perfectly matched those found in age-matched controls analyzed with the conventional “reference” method used in our diagnostic laboratory. This was quite unexpected, due to the following reasons:—DNA was extracted from differentially manipulated blood specimens (WB, PBMC, and dried blood) and using diverse methods (different buffers and reagents may potentially exert an effect on the end-result);—the amount of target DNA used for the two amplification protocols was different;—the methods of TREC and KREC evaluation were different (with qRT-PCR the values are extrapolated from a standard curve, while with dPCR the results are expressed as absolute values);—the mathematical formula used to calculate the number of TRECs and KRECs/mL of blood are diverse. In addition, it must be taken into account that the data were all obtained in different experimental procedures, in order to reproduce the “real world” conditions necessary for a test to be used for routine analyse. This introduces further variations in the results. Therefore, although the experiment performed with dilutions of plasmid DNA using dPCR allowed the detection of one copy of TRECs and one of KRECs, while 2.5 copies of the targets were detected by qRT-PCR, due to above reported differences, one cannot be confident enough to possibly state that dPCR is more sensitive than qRT-PCR for the quantification of TRECs and KRECs in peripheral blood. However, with qRT-PCR approach, the lowest levels of TRECs and KRECs are evidenced at Ct greater than 35. At values >35, the qRT-PCR-generated data have poor precision, are not always reproducible, and the method cannot be considered quantitative. Furthermore, we previously reported [7] that the detection limit for qRT-PCR method for TRECs was approximately 10 TRECs or KRECs copies and Lorenzi et al. [3] demonstrated that as the number of TRECs quantified per sample fell below 10, variations in the final result increased. Finally, an advantage of dPCR is that, when working at the limit of assay sensitivity, the probability of TREC and KREC detection could be easily increased by quantifying these targets from replicate aliquots of individual samples and adjusting the derived values accordingly.

To our knowledge, no data are available on KREC evaluation from adult dried blood samples, thus this is the first report in which both TRECs and KRECs have been simultaneously quantified starting from blood of adults adsorbed on FS.

## Conclusions

We demonstrated that FS-absorbed dried blood samples allow the detection of both TRECs and KRECs using dPCR in adult samples, including subjects over 70 s, notoriously known to present low number of TRECs, and also in those in whom these targets were undetectable when evaluated by qRT-PCR. The proposed method, based on combining DNA isolation from dried blood on FS followed by dPCR-based analysis, enables the quantification of nucleic acid targets without the need of using calibration curves and shows a high degree of efficiency, sensitivity, accuracy, and precision. Importantly, it represents a novel and valid option for studying the extent of newly produced T and B cells in adults and elderly population.

Future studies are needed to evaluate this methodology in the context of a larger cohort of adult patients with multiple sclerosis, rheumatoid arthritis or patients who underwent bone marrow transplantation, in order to monitor their immune status and their immune recovery during immunomodulating-immunosuppressive therapies.

## Additional files

**Additional file 1: Figure S1.** dPCR plots for TREC and KREC quantification. A) Representative dPCR plots of TREC and KREC quantification in HeLa cells. The cell line is used to establish the threshold values, which were set up at 5000 for FAM (TRECs) fluorescence and 4000 for VIC (KRECs) fluorescence. B) Representative dPCR plots of TREC and KREC quantification in a positive sample showing that the threshold values allow a precise separation between positive and negative dot plots. The data points in the plots are color-coded: FAM (blue), VIC (red), FAM plus VIC (green), undetermined (grey) and not amplified (yellow).

**Additional file 2: Table S1.** Intra-assay and inter-assay variation for dPCR.

**Additional file 3: Table S2.** TREC and KREC values obtained in different experimental procedures of qRT-PCR and dPCR.

**Additional file 4: Figure S2.** Levels of TRECs and KRECs in adults divided by age. Mean levels of TRECs and KRECs obtained by dPCR starting from DNA isolated from dried blood adsorbed on FS (red bars) and by qRT-PCR starting from DNA prepared from PBMC (blue bars) in the indicated age groups of adults. Error bars represent standard deviations.

## Abbreviations

dPCR: digital PCR
FS: flocked swabs
KRECs: K-deleting recombination excision circles
PBMC: peripheral blood mononuclear cells
qRT-PCR: quantitative real time PCR
TCR: T-cell receptor
TRECs: TCR excision circles
WB: whole blood
