# Supplementary figures and images for: Detection of newly produced T and B lymphocytes by digital PCR in blood stored dry on nylon flocked swabs
Source: J Transl Med. 2017 Apr 5;15:70. doi: 10.1186/s12967-017-1169-9 (PMC5381048; doi:10.1186/s12967-017-1169-9)

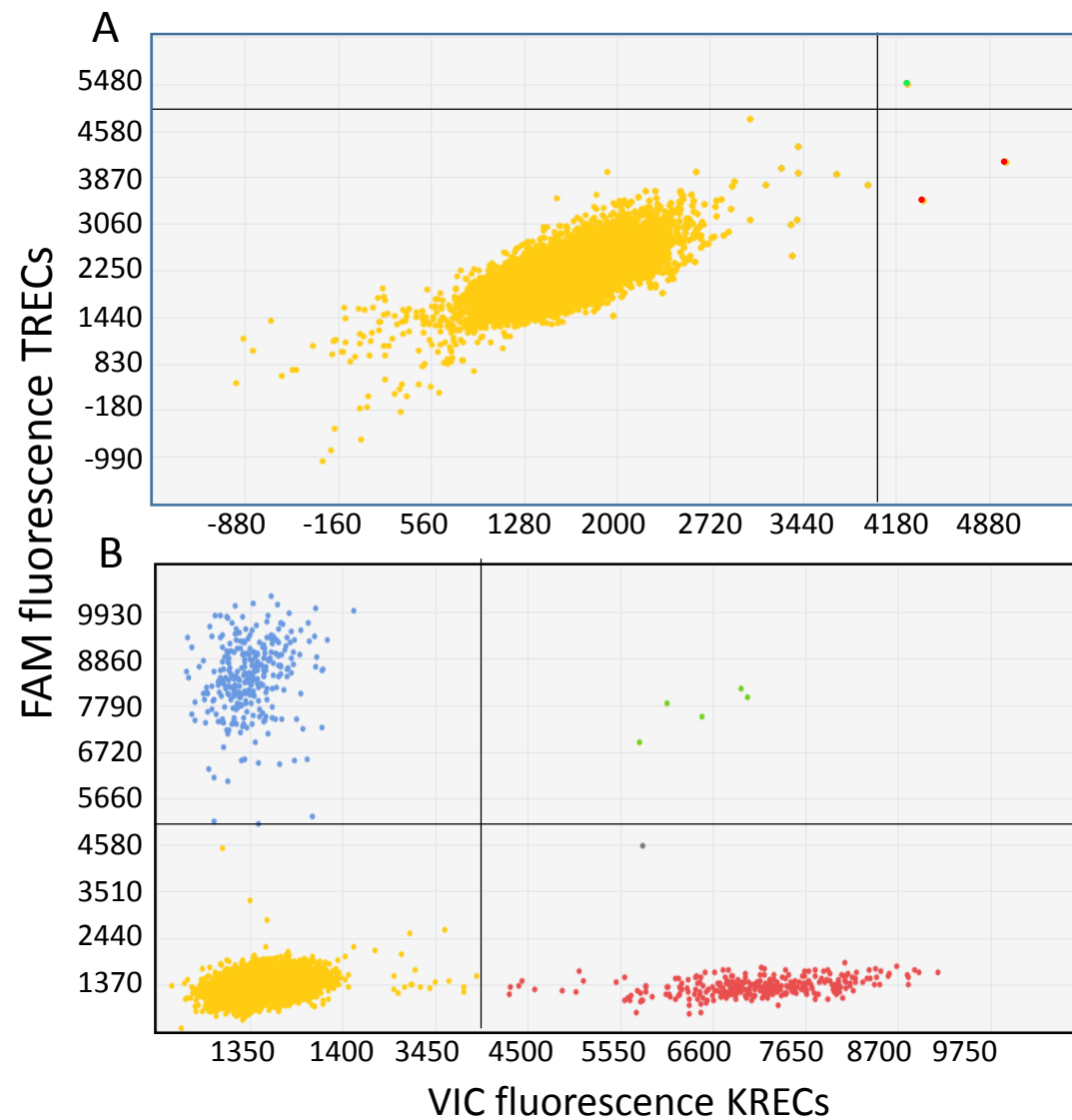

Supplement: Supplementary file 1 — Additional file 1: Figure S1. dPCR plots for TREC and KREC quantification. A) Representative dPCR plots of TREC and KREC quantification in HeLa cells. The cell line is used to establish the threshold values, which were set up at 5000 for FAM (TRECs) fluorescence and 4000 for VIC (KRECs) fluorescence. B) Representative dPCR plots of TREC and KREC quantification in a positive sample showing that the threshold values allow a precise separation between positive and negative dot plots. The data points in the plots are color-coded: FAM (blue), VIC (red), FAM plus VIC (green), undetermined (grey) and not amplified (yellow). [file 12967_2017_1169_MOESM1_ESM.pdf]

Additional File 3: Fig. S2

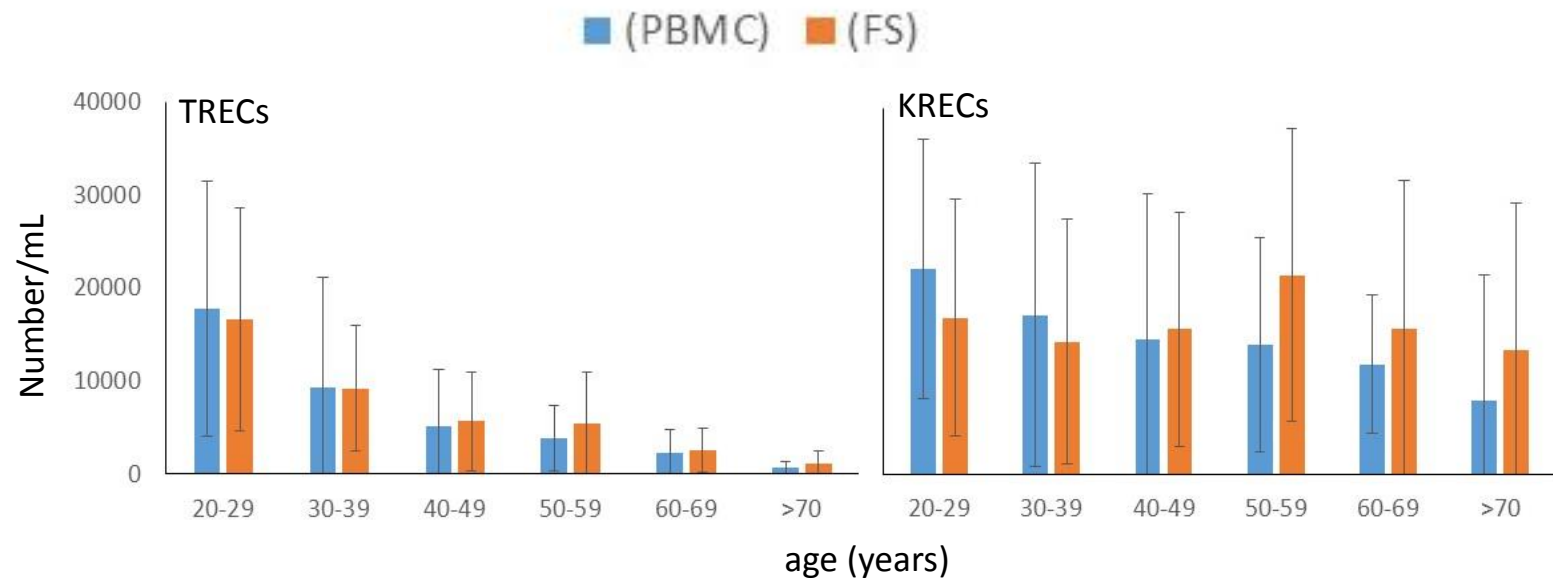

Supplement: Supplementary file 4 — Additional file 4: Figure S2. Levels of TRECs and KRECs in adults divided by age. Mean levels of TRECs and KRECs obtained by dPCR starting from DNA isolated from dried blood adsorbed on FS (red bars) and by qRT-PCR starting from DNA prepared from PBMC (blue bars) in the indicated age groups of adults. Error bars represent standard deviations. [file 12967_2017_1169_MOESM4_ESM.pdf]
