# Supplementary material for: Detection of newly produced T and B lymphocytes by digital PCR in blood stored dry on nylon flocked swabs
Source: J Transl Med. 2017 Apr 5;15:70. doi: 10.1186/s12967-017-1169-9 (PMC5381048; doi:10.1186/s12967-017-1169-9)
Supplement: Supplementary file 2 — Additional file 2: Table S1. Intra-assay and inter-assay variation for dPCR. [file 12967_2017_1169_MOESM2_ESM.pdf]

Table S1. Intra-assay and inter-assay variation for dPCR.

		Mean	standard deviation	% CV
Intra-assay	TRECs	6744	720	11
	KRECs	19653	1664	8
Inter-assay	TRECs	7522	1004	13
	KRECs	19758	2943	15
