# Supplementary material for: Detection of newly produced T and B lymphocytes by digital PCR in blood stored dry on nylon flocked swabs
Source: J Transl Med. 2017 Apr 5;15:70. doi: 10.1186/s12967-017-1169-9 (PMC5381048; doi:10.1186/s12967-017-1169-9)
Supplement: Supplementary file 3 — Additional file 3: Table S2. TREC and KREC values obtained in different experimental procedures of qRT-PCR and dPCR. [file 12967_2017_1169_MOESM3_ESM.docx]

| Table 2S. TRECs and KRECs values obtained in different experimental procedures of qRT-PCR | | | | | | | | | |
| --- | --- | --- | --- | --- | --- | --- | --- | --- | --- |
| and dPCR. |  |  |  |  |  |  |  |  |  |
|  | **TRECs** | | | |  | **KRECs** | | | |
|  | **qRT-PCR^a^** | | **dPCR** | |  | **qRT-PCR** | | **dPCR** | |
| Replicates | μg | mL | μg | mL |  | μg | mL | μg | mL |
| 1 | und | und | 19 | 751 |  | und | und | 22 | 1 423 |
| 2 | und | und | 18 | 705 |  | und | und | 23 | 1 554 |
| 3 | und | und | 18 | 710 |  | und | und | 20 | 1351 |
| 4 | und | und | 18 | 689 |  | und | und | 20 | 1316 |
| 5 | und | und | 18 | 715 |  | und | und | 20 | 1307 |
| ^a^qRT-PCR: quantitative real time PCR; dPCR: digital PCR; und: undetectable | | | | | | | | |  |
